# Impact of setup errors on the robustness of linac‐based single‐isocenter coplanar and non‐coplanar VMAT plans for multiple brain metastases

**DOI:** 10.1002/acm2.14317

**Published:** 2024-03-04

**Authors:** Xiaohuan Sun, Fada Guan, Qinghui Yun, Matthew Jennings, Simon Biggs, Zhongfei Wang, Wei Wang, Te Zhang, Mei Shi, Lina Zhao

**Affiliations:** ^1^ Department of Radiation Oncology Xijing Hospital, Air Force Medical University Xi'an China; ^2^ Department of Therapeutic Radiology Yale University School of Medicine New Haven Connecticut USA; ^3^ Department of Equipment Xijing Hospital, Air Force Medical University Xi'an China; ^4^ Department of Medical Physics Townsville University Hospital Douglas Queensland Australia; ^5^ Radiotherapy AI Pty Ltd Wagga Wagga Australia

**Keywords:** coplanar and noncoplanar VMAT plans, multiple brain metastases, patient setup errors, single isocenter

## Abstract

**Purpose:**

Patient setup errors have been a primary concern impacting the dose delivery accuracy in radiation therapy. A robust treatment plan might mitigate the effects of patient setup errors. In this reported study, we aimed to evaluate the impact of translational and rotational errors on the robustness of linac‐based, single‐isocenter, coplanar, and non‐coplanar volumetric modulated arc therapy treatment plans for multiple brain metastases.

**Methods:**

Fifteen patients were retrospectively selected for this study with a combined total of 49 gross tumor volumes (GTVs). Single‐isocenter coplanar and non‐coplanar plans were generated first with a prescribed dose of 40 Gy in 5 fractions or 42 Gy in 7 fractions to cover 95% of planning target volume (PTV). Next, four setup errors (+1  and +2 mm translation, and +1° and +2° rotation) were applied individually to generate modified plans. Different plan quality evaluation metrics were compared between coplanar and non‐coplanar plans. 3D gamma analysis (3%/2 mm) was performed to compare the modified plans (+2 mm and +2° only) and the original plans. Paired *t*‐test was conducted for statistical analysis.

**Results:**

After applying setup errors, variations of all plan evaluation metrics were similar (*p* > 0.05). The worst case for V100% to GTV was 92.07% ± 6.13% in the case of +2 mm translational error. 3D gamma pass rates were > 90% for both coplanar (+2 mm and +2°) and the +2 mm non‐coplanar groups but was 87.40% ± 6.89% for the +2° non‐coplanar group.

**Conclusion:**

Translational errors have a greater impact on PTV and GTV dose coverage for both planning methods. Rotational errors have a greater negative impact on gamma pass rates of non‐coplanar plans. Plan evaluation metrics after applying setup errors showed that both coplanar and non‐coplanar plans were robust and clinically acceptable.

## 1 INTRODUCTION

A very high proportion of cancer patients develop brain metastases in their tumor history, and approximately half of the patients dying from cancer have been found to have brain metastases at autopsy.^1^ Particularly, over two‐thirds of brain metastasis cases have multiple lesions. Radiotherapy is one of the most effective modalities to treat multiple brain metastases (MBM). Currently, major radiotherapy facility for treating brain metastases include Gamma Knife (Elekta, Stockholm, Sweden), CyberKnife (Accuray, Sunnyvale, California, USA), TomoTherapy (Accuray, Sunnyvale, California, USA), and the modern medical linear accelerator (linac).^2^, ^3^ Each modality has its own advantages and limitations. Linacs are the most used modality due to the wide range of disease sites with which they are suitable to treat. The advent of the volumetric modulated arc therapy (VMAT) technique has facilitated the use of linacs for challenging cases, including MBM.

Typically, linac‐based treatment for MBM adopts the strategy of dose delivery with multiple isocenters in which each isocenter is assigned to a single lesion in the treatment plan. Consequently, patients need to be repositioned for the setup of each isocenter during treatment, which considerably increases treatment times. Moreover, multiple intra‐fractional repositioning also increases the risk of mistreatment and overall uncertainty in dose delivery. In contrast, the primary advantage of a single‐isocenter treatment is that it provides simultaneous treatment for MBM and therefore can mitigate the disadvantages of using multiple isocenters.

Hypo‐fractionated stereotactic radiosurgery (SRS) and stereotactic radiation therapy (SRT) are often used to treat MBM due to the high local control rates.^4^, ^5^, ^6^, ^7^, ^8^, ^9^, ^10^ There are several unique characteristics of SRS/SRT for treating MBM such as high dose per fraction and a small number of fractions to increase the biologically effective dose compared to the conventional dose fractionation, and steep dose gradient to spare normal tissues/organs. The study by Kraft et al. found that SRS/SRT using single‐isocenter VMAT for MBM achieved high local control rates in spite of the distance of a disease site to the isocenter.^11^ Nevertheless, there are also more challenges to clinically implement the single‐isocenter VMAT‐based SRS/SRT. For example, multiple gross tumor volumes (GTVs) may lie a significant distance off‐axis, where the dosimetric effect of rotational errors might be amplified. Therefore, it is critical to evaluate the robustness of a treatment plan by considering the possible setup errors, particularly important for an SRS/SRT. A robust plan should provide a high dose coverage to the targets and spare the organs‐at‐risk (OARs) within the tolerance after different setup errors are introduced.

In the current clinical practice, two different types of VMAT planning techniques are commonly used in the intracranial SRS/SRT: coplanar and non‐coplanar plans. Non‐coplanar plans may improve target dose coverage and OAR sparing relative to coplanar plans. However, when delivering a non‐coplanar plan, the intra‐fractional couch rotation increases treatment time and may introduce new setup errors. Many previous studies only investigated the robustness of non‐coplanar VMAT plans impacted by patient setup discrepancies.^5^, ^6^, ^7^, ^12^, ^13^ However, the studies comparing the impacts of setup errors on the robustness of coplanar and non‐coplanar plans are scarce.

In this reported study, we aimed to (1) evaluate and compare the plan quality of coplanar and non‐coplanar approaches for single‐isocenter plans in the treatment of multiple brain metastases, and (2) evaluate and compare the impact of translational and rotational setup errors on the robustness of these two planning approaches.

## 2 METHODS

### 2.1 Patient selection

Fifteen MBM patients were retrospectively recruited in this study. These patients were treated between January 2018 and January 2022 in the Department of Radiation Oncology at XXX hospital. The basic patient characteristics are summarized in Table S1 in the Supplementary Materials.

### 2.2 CT simulation and target delineation

All patients were scanned using the Brilliance CT Big Bore (Philips Healthcare, Best, Netherlands) system in a head‐first‐supine orientation with a thermoplastic mask for immobilization during the simulation. The CT images were acquired with 2 mm slice thickness and were then used for target delineation and dose calculation during the treatment planning. Fused MR images on the CT images were used to aid target delineation. The planning target volume (PTV) was then derived by adding an isotropic 2 mm expansion from the GTV. All GTV expansions were combined to give the single PTV.

### 2.3 Treatment planning of original coplanar and non‐coplanar plans

For each patient, a coplanar and a non‐coplanar VMAT treatment plan were created as the original reference plans. All the plans were generated using the treatment planning system Eclipse (Varian Medical Systems, Version 13.5) and calculated using the anisotropic analytical algorithm in Eclipse. A 2 mm grid resolution was applied for dose calculation. A 6‐MV photon beam with a maximum dose rate of 600 MU/min was selected for all treatment plans. A Varian Clinac iX linac (Varian Medical Systems, Palo Alto, California, USA) was used to treat patients clinically. The prescribed dose was 40 Gy in 5 fractions or 42 Gy in seven fractions. All plans were normalized to make 95% of the PTV receive 100% of the prescribed dose. The dose constraints for OARs followed the guidance in Task Group Report 101 of the American Association of Physicists in Medicine (AAPM).^14^

The single isocenter was set at the approximate midpoint of the centroids of all GTVs. The coplanar plan was generated using three to four VMAT arc beams. The non‐coplanar plan was generated with the following arc arrangement: one or two full 360° arcs (couch angle at 0°) and two half arcs (couch angles at 45° and 315°, or at 60° and 300°). The collimator angles were adjusted based on the shape of the targets.

### 2.4 Simulation of patient setup errors and generation of corresponding plans

To evaluate the impact of patient setup errors on patient dose distribution, four hypothetical setup errors were applied to the original coplanar and non‐coplanar plans. The setup errors included two translational errors and two rotational errors. When applying the translational error, the isocenter shift was set to +1  or +2 mm individually for all the three directions in Eclipse for the original plan. When applying the rotational error, the rotational angle was set to +1° or +2° individually for the roll, yaw, and pitch axes. The CT set was first exported from Eclipse in DICOM RT format and then imported into the software MIM Maestro (version 7.0.5; MIM Software Inc, Beachwood, Ohio, USA). The CT set was rotated in MIM in accordance with rotational setup error, then the newly generated rotated CT DICOM file from MIM was imported back to Eclipse. All structures in the reference CT DICOM file were copied to the rotated CT DICOM file. Both original coplanar and non‐coplanar plans were replicated to new plans using the same treatment parameters [such as monitor units (MUs), multi‐leaf collimator (MLC) patterns, and arc geometry]. The plan dose was re‐calculated by keeping the original MUs to generate the new plan. In total, for each original coplanar or non‐coplanar plan, four new plans were generated after applying the setup errors individually (+1 , +2 mm, +1°, and +2°), as listed in Table S2 in the Supplementary Materials.

### 2.5 Plan quality and robustness evaluation metrics

Different dose‐volume metrics were used to evaluate the plan quality and robustness. In the original plan, the conformity index (CI) and dose gradient index (GI) of the PTV were calculated as the plan evaluation metrics to compare between coplanar and non‐coplanar plans. The CI was calculated using the formula proposed by Paddick^15^ as shown in Equation (1):

(1) $$CI\;=\frac{TV_{PIV}^{2}}{TV\times PIV}\;,$$

where *TV* is the target volume (here it is volume of PTV), *PIV* stands for prescription isodose volume and it is the volume covered by the prescription isodose line, and *TV_PIV_* is the overlapped volume (intersection) of TV and PIV. *CI* ranges between 0 and 1.0 and the ideal dose coverage is CI = 1.0.

The GI was calculated using the formula proposed by Paddick^16^ as shown in Equation (2):

(2) $$GI\;=\frac{PV_{50\%}}{PIV}\;,$$

where PV_50%_ is the volume covered by the 50% prescription isodose line, and PIV is the volume covered by the prescription isodose line. GI value is > 1, and a smaller GI means a steeper dose gradient. A small GI is preferred to spare the OARs.

For OARs, mean dose to the whole brain, V4 (volume of the brain receiving 4 Gy or higher) and V12 (volume of the brain receiving 12 Gy or higher) of the whole brain, maximum doses (D_max_) of the brain stem, left and right eyes, left and right eye lens, and optic pathways were calculated.

After applying the setup errors, in addition to the dose metrics to OARs, the V100(%) of PTV (percentage of PTV receiving > = 100% prescribed dose) and V100(%) of GTV (percentage of GTV receiving > = 100% prescribed dose) were calculated. Notably, in the original plans, dose was normalized to make 100% of prescribed dose cover 95% of PTV; therefore, V100(%) of PTV was equal to 95% for all original plans. However, after applying setup errors, the V100(%) of PTV was expected to change. By subtracting the V100(%) of PTV/GTV in the original plan groups, the variations of V100(%) after applying setup errors were calculated and compared between coplanar and non‐coplanar plans.

3D gamma analysis was performed to compare the modified plan with a setup error (as the evaluation plan) and the original plan (as the reference plan) using the open‐source software PyMedPhys (version 0.39.3)^17^ (https://pypi.org/project/pymedphys/). The dose threshold value was set to 10% of the D_max_ in the original plan for gamma index calculation. The criteria (3%/2 mm) were used in the gamma analysis, as recommend by the AAPM Task Group Report 218 for patient‐specific quality assurance.^18^

### 2.6 Statistical analysis

The IBM SPSS Statistics 26.0 software was used for statistical analysis. A paired *t*‐test using a confidence threshold of *p* < 0.05 was performed for all comparisons. The Origin software (version 2021b) was used for data analysis and plotting. In the plots, outlier data points were marked explicitly. Outliers of a dataset were those data points outside the range of 1.5 × interquartile range (IQR) from the first quartile and the third quartile.

## 3 RESULTS

The results were all expressed in the form of mean ± SD (standard deviation). *p* < 0.05 indicated there was a significant difference between the mean values from the two comparison groups. For PTV related quantities, the sample size was *n *= 15, the number of patients in this study. For GTV related quantities, the sample size was *n *= 49, the number of GTVs assessed across all patients.

### 3.1 Comparison of original coplanar and non‐coplanar plans

**FIGURE 1:**
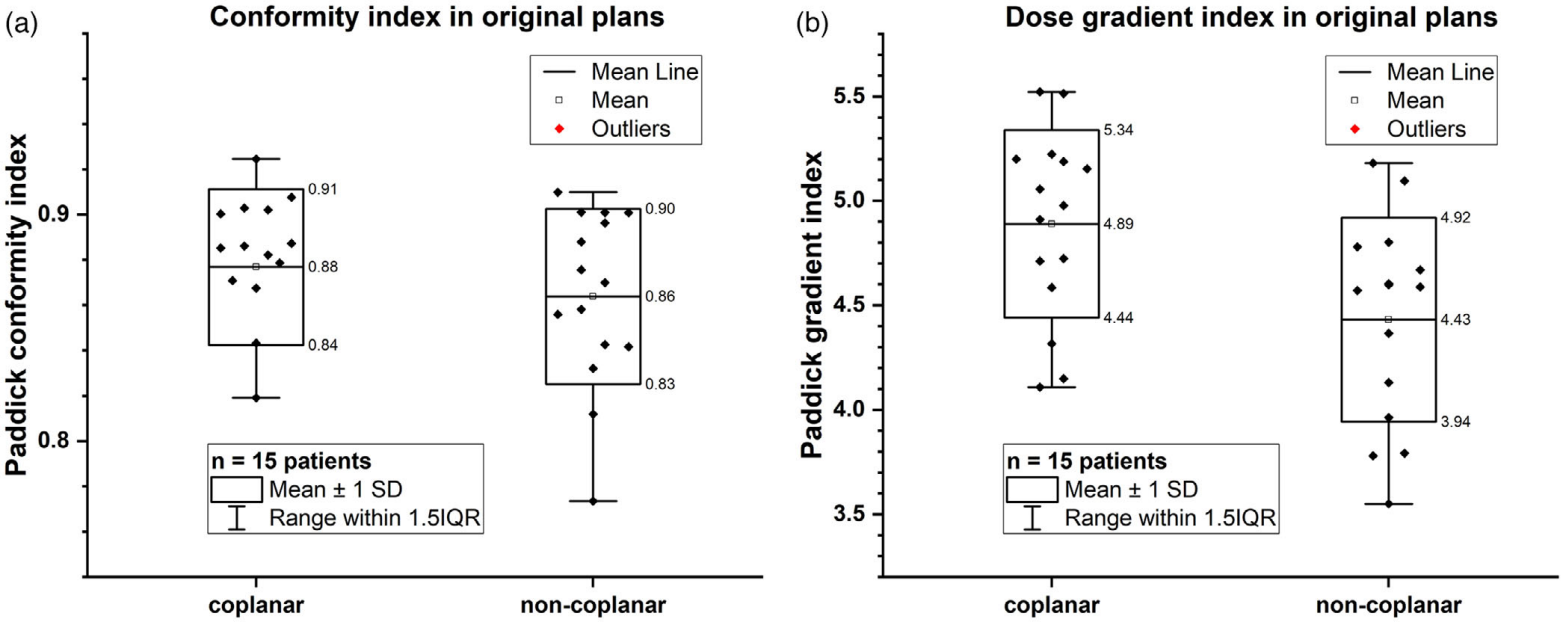
(a) Comparison of Paddick conformity index (a) and Paddick dose gradient index (b) of the PTV between the original coplanar and non‐coplanar plans.

The CI and GI of PTV from the original coplanar and non‐coplanar plans for all the 15 patients are illustrated in Figure 1a and 1b. The CI of the original coplanar and non‐coplanar plan group was 0.88 ± 0.03 and 0.86 ± 0.01, listed in Table S3 in the Supplementary Materials. The *t*‐test showed that there was a significant difference (*p *= 0.002) in CI between coplanar and non‐coplanar plans, and the coplanar method was superior in dose conformity, indicated by its higher CI. Nevertheless, the numerical difference of CI between these two groups was small. The GI of the original coplanar and non‐coplanar plan group was 4.89 ± 0.45 and 4.43 ± 0.49, respectively (see Table S3). The *t*‐test result showed that there was a significant difference (*p *< 0.001) in GI between coplanar and non‐coplanar plans, and the non‐coplanar method was superior in dose gradient, indicated by its lower GI.

Table S3 also depicts dosimetric evaluation parameters of OARs for both the original coplanar and non‐coplanar plans. The whole brain V12 was 17.10% ± 8.38% and 14.79% ± 8.93% for the coplanar and non‐coplanar plans, respectively. There was a significant difference between these two groups in the whole brain V12 (*p *= 0.002) and the non‐coplanar method was superior as indicated by its lower volume coverage. *D*_max_ to the optic chiasma was 4.90  ± 3.44  and 5.95 Gy ± 3.36 Gy for the coplanar and non‐coplanar plans, respectively. The *t*‐test result showed a significant difference in *D*_max_ to the optic chiasma (*p *= 0.041), where the coplanar method was superior. For all other dosimetric evaluation parameters, there were no significant differences between the coplanar and non‐coplanar groups.

### 3.2 Comparison of the impact of translational errors on coplanar and non‐coplanar plans

When the patient setup error is introduced, the clinical concern is more about whether the GTV can still receive the sufficiently high dose coverage. Therefore, when the setup error was introduced in our study, the CI and GI of PTV were no longer evaluated in the modified plans. Instead, the V100% of PTV and GTV were evaluated as the metrics and their variations to the original plans were used to evaluate the plan robustness. The V100% of PTV (marked as PTV100%) of the original plans were equal to 95% in all original plans as planed dose distributions were normalized to make prescribed dose cover 95% of the PTV.

**FIGURE 2:**
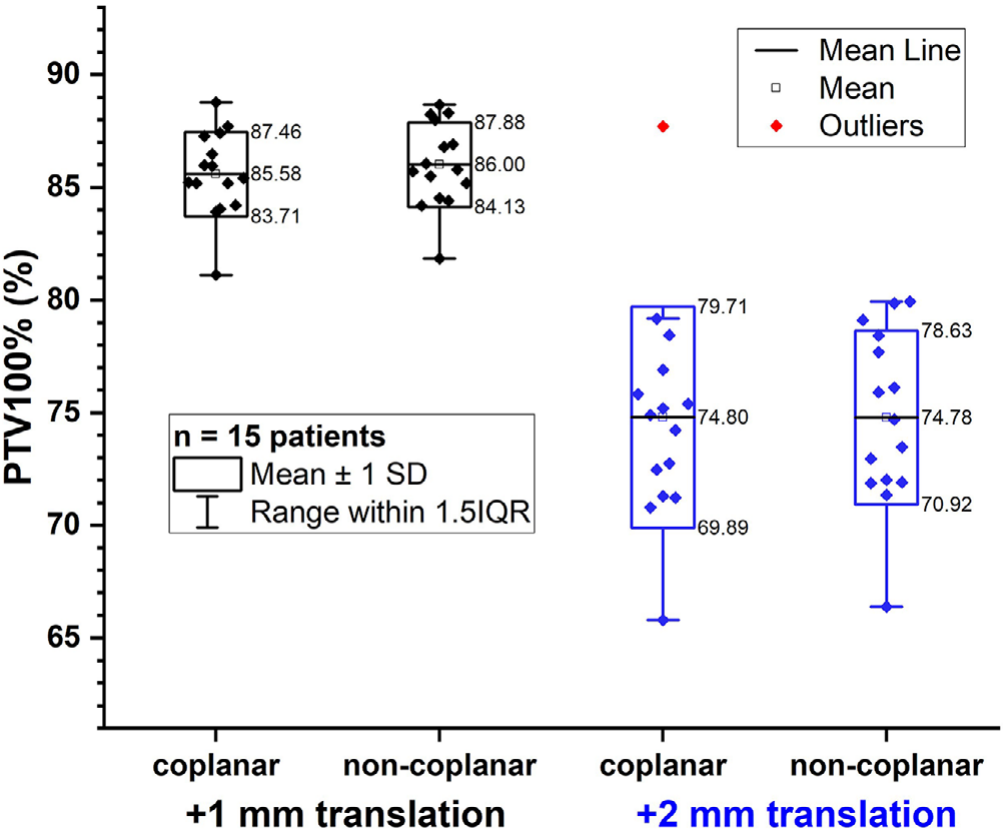
Comparison of PTV100%(%) between the coplanar and non‐coplanar plans with +1  and +2 mm translational error, respectively.

The PTV100% distributions of modified plans with translational errors from coplanar and non‐coplanar plans were compared in Figure 2. After applying +1 mm translational error, the variations of PTV100% were −9.42% ± 1.88% and −9.00% ± 1.88% for the coplanar and non‐coplanar plans, respectively. After applying a +2 mm translational error, the corresponding variations were −20.20% ± 4.91% and −20.22% ± 3.85%. There were no significant differences between the coplanar and non‐coplanar groups after applying the translational errors (see Table S4 in the Supplementary Materials).

**FIGURE 3:**
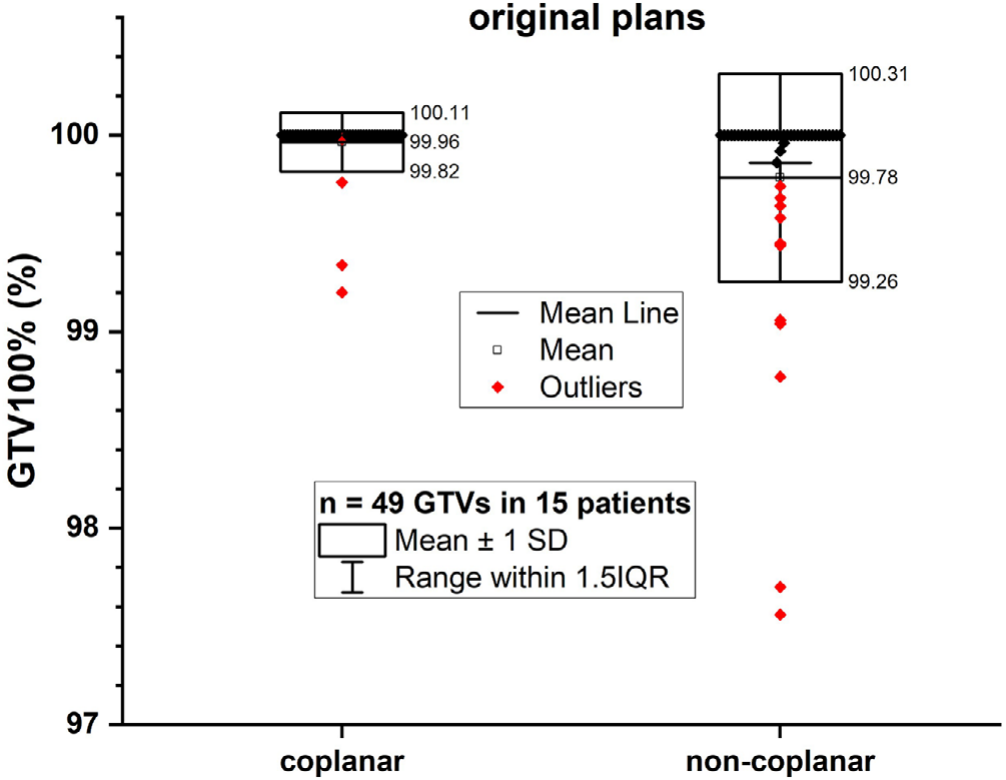
Comparison of GTV100%(%) between the original coplanar and non‐coplanar plans.

**FIGURE 4:**
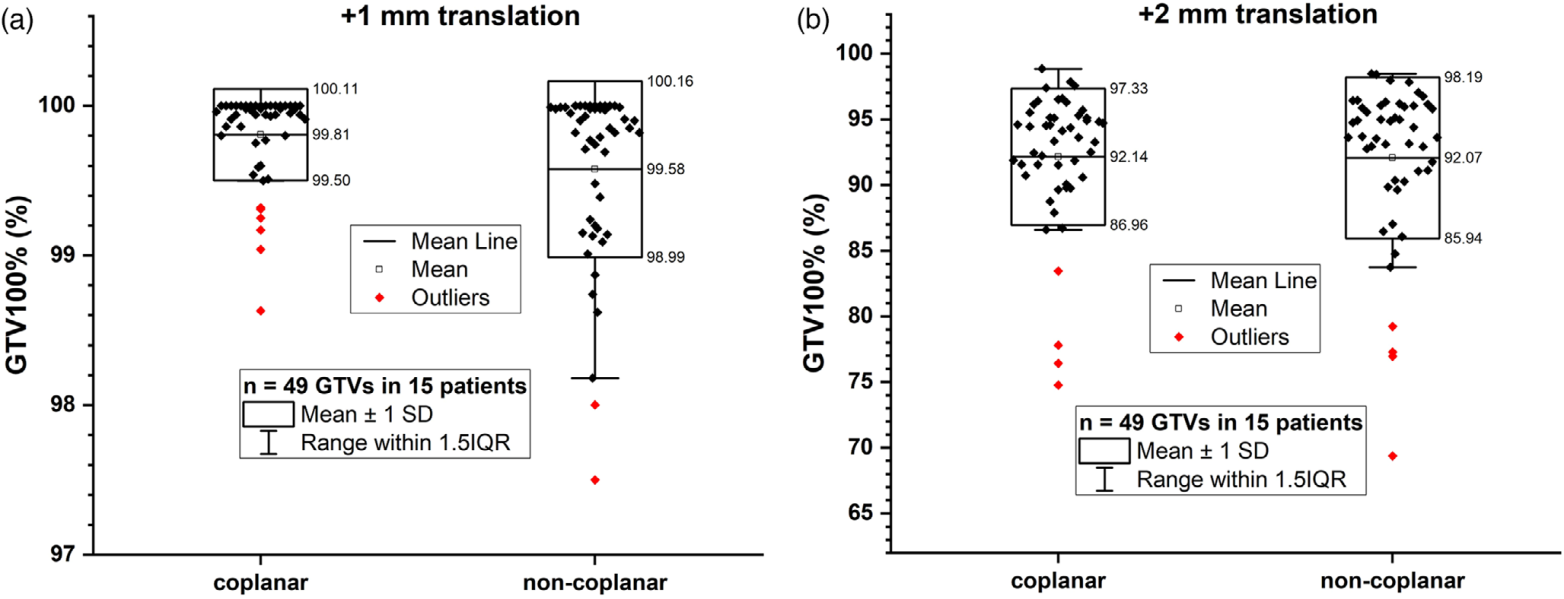
Comparison of GTV100%(%) between the coplanar and non‐coplanar plans with +1 mm (a) and +2 mm (b) translational error, respectively.

The distributions of V100% of GTV (marked as GTV100%, n = 49) of the original plans were compared in Figure 3. As listed in Table S4, the GTV100% was 99.96% ± 0.15% and 99.78% ± 0.53% for the original coplanar group and the non‐coplanar group, respectively. Although the numerical values of GTV100% of original coplanar plans and non‐coplanar plans were close to each other, the t‐test result showed a significant difference (*p *= 0.019). The GTV100% distributions of modified plans with translational errors from coplanar and non‐coplanar plans were compared in Figure 4. After applying +1 mm translational error, the variations of GTV100% were −0.16% ± 0.29% and −0.21% ± 0.43% for coplanar and non‐coplanar plans. After applying +2 mm translational error, the corresponding variations were −7.82% ± 5.20% and −7.72% ± 6.19%. There were no significant differences between the coplanar and non‐coplanar groups after applying the translational errors (*p *> 0.05), listed in Table S4.

As listed in Table S4, the dose‐volume evaluation metrics of the OARs did not have large variations after applying translational errors. The V4 and V12 to the whole brain and the *D*_max_ to most of other OARs were reduced. The only large increase in *D*_max_ was to the left lens after applying the +2 mm translational error. The dose increase was 1.20  ± 1.78  and 1.54 Gy ± 1.85 Gy for the coplanar and non‐coplanar groups, respectively. Nevertheless, the dose in the modified plans was still within the tolerance to lens. The variations of dose evaluation metrics of the OARs did not have significant differences between the coplanar and non‐coplanar groups (see Table S4).

### 3.3 Comparison of the impacts of rotational errors on coplanar and non‐coplanar plans

**FIGURE 5:**
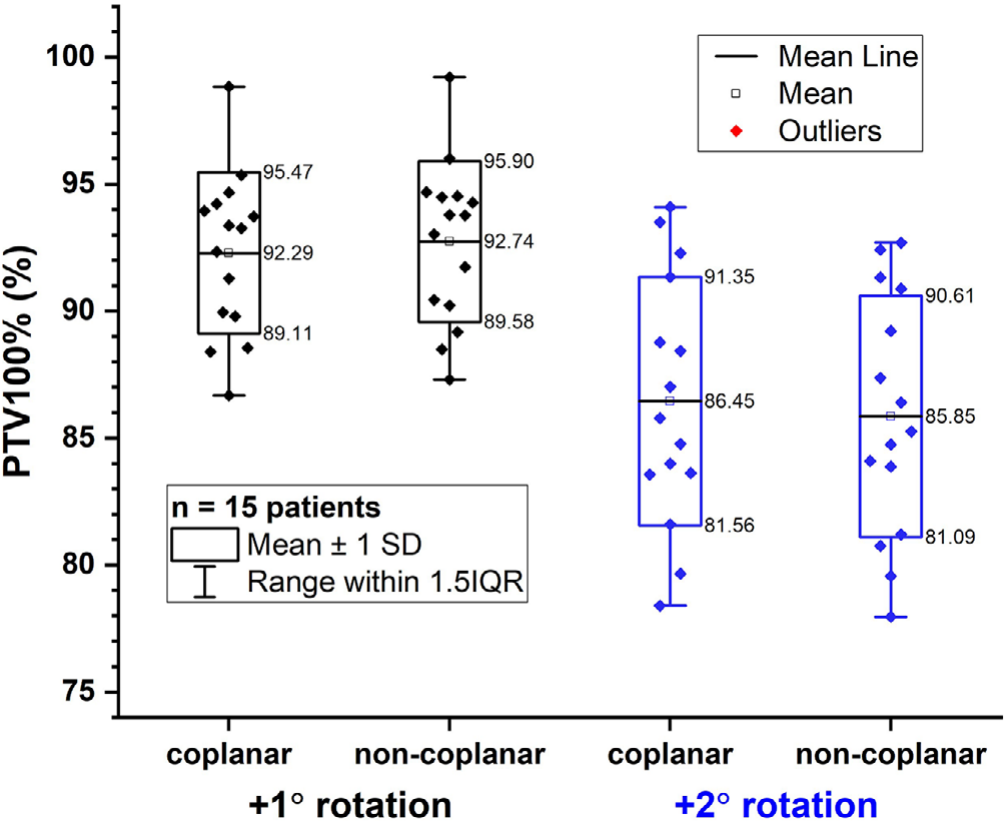
Comparison of PTV100%(%) between the coplanar and non‐coplanar plans with +1° and +2° rotational error, respectively.

The PTV100% distributions of modified plans with rotational errors from coplanar and non‐coplanar plans were compared in Figure 5. After applying +1° rotational error, the variations of PTV100% were −2.71% ± 3.19% and −2.26% ± 3.16% for coplanar and non‐coplanar plans. After applying +2° rotational error, the corresponding variations were −8.55% ± 4.90% and −9.15% ± 4.76%. There were no significant differences between the coplanar and non‐coplanar groups after applying the rotational errors (see Table S5 in the Supplementary Materials).

**FIGURE 6:**
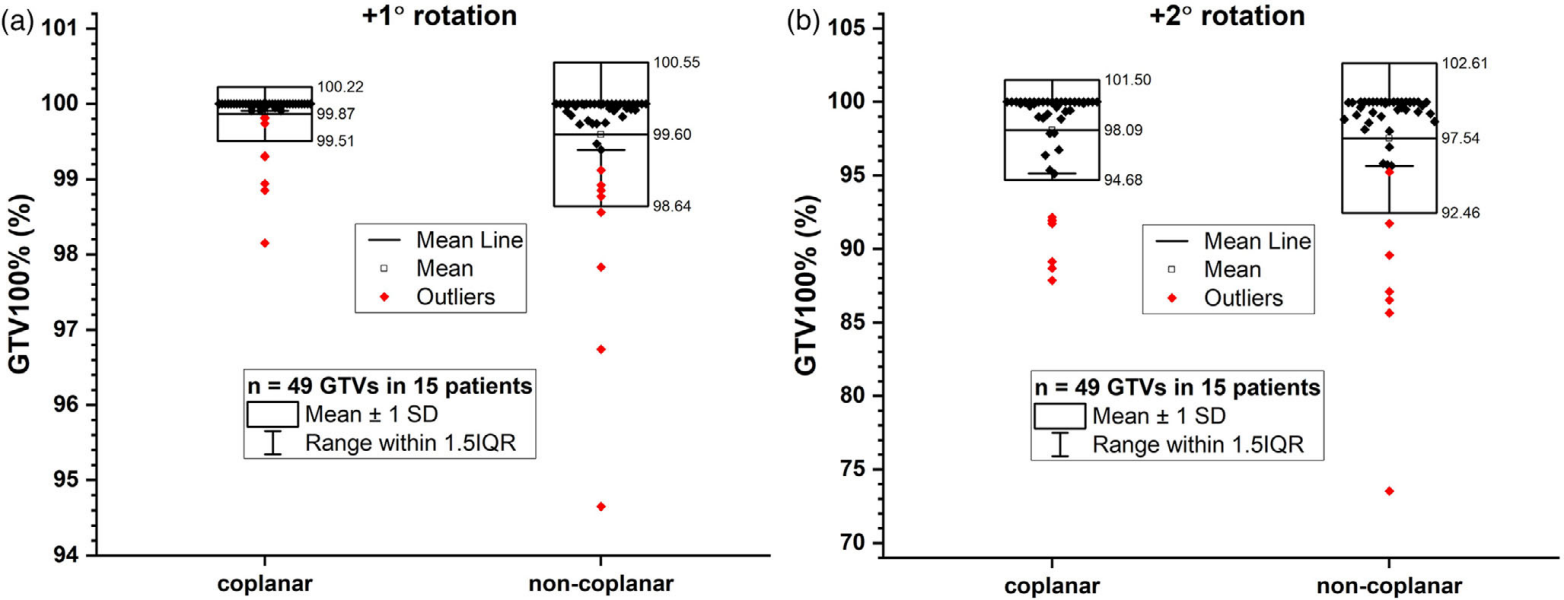
Comparison of GTV100%(%) between the coplanar and non‐coplanar plans with +1° (a) and +2° (b) rotational error, respectively.

The GTV100% distributions of modified plans with rotational errors from coplanar and non‐coplanar plans were compared in Figure 6. After applying +1° rotational error, the variations of GTV100% were −0.10% ± 0.33% and −0.19% ± 0.81% for coplanar and non‐coplanar plans. After applying +2° rotational error, the corresponding variations were −1.88% ± 3.42% and −2.25% ± 4.94%. There were no significant differences between the coplanar and non‐coplanar groups after applying the rotational errors (see Table S5).

As listed in Table S5, the dose‐volume evaluation metrics of the OARs did not have large variations after applying rotational errors. The maximum dose increase of *D*_max_ was below 0.5 Gy, and the *D*_max_ to OARs in the modified plans was still with the tolerance. The variations of dose evaluation metrics of the OARs did not have significant differences between the coplanar and non‐coplanar groups (*p *> 0.05), listed in Table S5.

The original coplanar and non‐coplanar plans of patient #8 in our study and the modified plans with different setup errors were compared in Figure S1 in the Supplementary Materials.

### 3.4 3D gamma analysis

**FIGURE 7:**
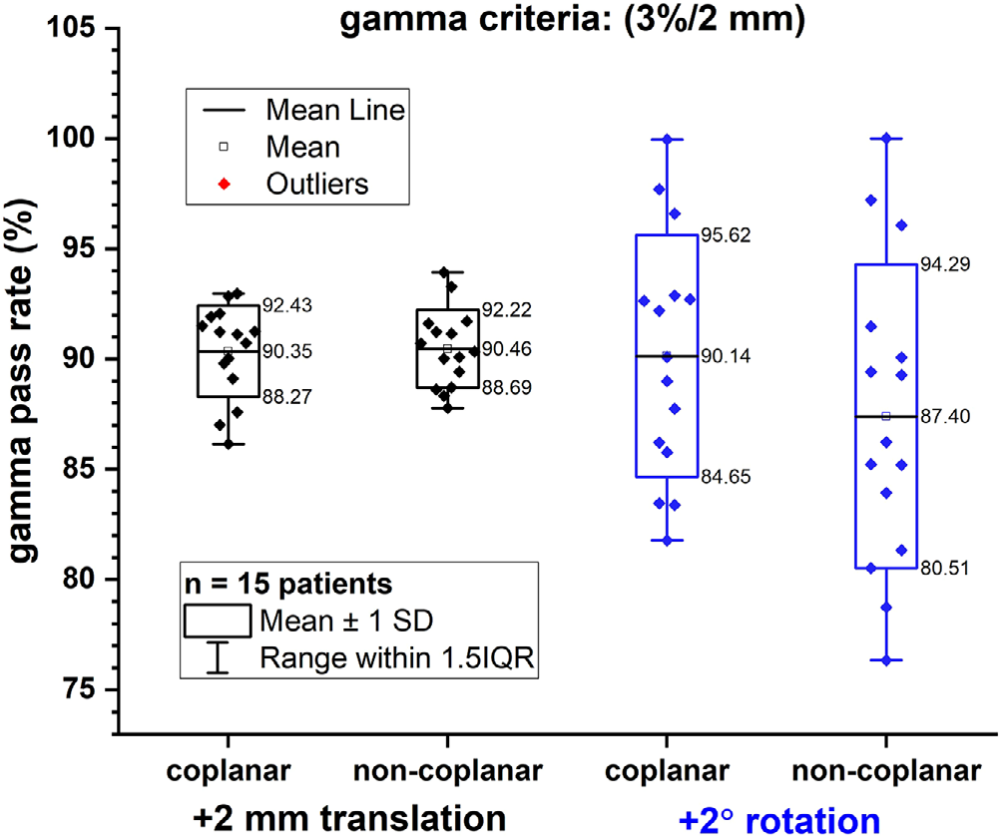
Distributions of 3D gamma analysis pass rate of the modified plans with +2 mm translational error and +2° rotational error for coplanar and non‐coplanar plans.

Only the plans with +2 mm translational error and +2° rotational error were performed with the 3D gamma analysis. The results of gamma pass rate were listed in Table S6. After applying the +2 mm translational error, the gamma pass rate was 90.35 % ± 2.08% and 90.46% ± 1.76% for coplanar and non‐coplanar groups, respectively. After applying the +2° rotational error, the gamma pass rate was 90.14% ± 5.49% and 87.40% ± 6.89% for coplanar and non‐coplanar groups, respectively. The +2° rotational error scenario failed the 3D gamma analysis. The distributions of 3D gamma pass rate of modified plans are shown in Figure 7. See Figure S2–S9 in the Supplementary Materials for the results of the 3‐D gamma comparisons between coplanar and non‐coplanar plans for patient #8.

## 4 DISCUSSION

Because of its simplicity in patient setup, the single‐isocenter method has been increasingly used in LINAC‐based treatment for multiple brain metastases. Kraft et al. concluded that SRS/SRT using single‐isocenter VMAT for multiple targets achieved high local control rates for metastases in spite of the distance of a disease site to the isocenter.^11^ The dose evaluation metrics for both targets and OARs of single‐isocenter plans in our study have shown that these plans are clinically acceptable.

Although the introduction of image guidance has greatly improved the patient setup accuracy and the subsequent dosimetric accuracy, it is still challenging to avoid setup errors in the whole workflow of radiation therapy. Therefore, it is critical to evaluate the robustness of a treatment plan by considering the possible setup errors. A robust plan should provide a high dose coverage to the targets and spare the OARs within the tolerance after different setup errors are introduced. Tsui et al. have studied the robustness of single isocenter and multi‐isocenter plans when different hypothetical setup errors were applied, but their single‐isocenter plans were all limited to the non‐coplanar design.^7^ Yoon et al. performed a study to evaluate the combined effect of dose gradient and rotational error on prescribed dose coverage for single isocenter multiple brain metastases in frameless stereotactic radiotherapy. They concluded that in the dose gradient alongside the margin addition can be adjusted as an ancillary parameter for small targets to increase target coverage or at least limit the coverage reduction with possible patient positioning error.^12^ Notably, their work was limited to non‐coplanar plans. Prentou et al. studied the dosimetric impact of rotational errors on the VMAT plan quality of SRS for treating multiple brain metastases.^13^ They compared the single‐ and two‐isocenter treatment planning techniques. They found that introducing additional isocenter(s) appeared to partly mitigate severe target underdosage when limiting the lesion‐to‐isocenter distance to ≤ 4 cm, especially for much smaller targets in SRS. Like other studies, the study by Prentou et al. only involved non‐coplanar plans.

Our study comprehensively compared plan quality and evaluated robustness for both coplanar and non‐coplanar techniques. The results of dose‐volume plan evaluation metrics after applying the hypothetical setup errors have shown that no significant statistical differences were found between these two different planning methods. Furthermore, our results indicated that using either coplanar or non‐coplanar techniques to make single‐isocenter VMAT plans for multiple brain metastases can produce clinically acceptable, robust plans. Notably, although the GI of the non‐coplanar plan group was smaller (implying a steeper dose gradient), as listed in Table S2, the plan robustness did not deteriorate.

Clinical challenges using the non‐coplanar technique include not only the increase of treatment time, but also the increased risk of introducing patient setup errors. Intra‐fractional setup errors for non‐coplanar treatments can be mitigated using non‐coplanar image guidance, such as ExacTrac (Brainlab, Munich, Germany). However, not all the linacs are equipped with such imaging system. Our results have quantitatively shown that the setup errors can greatly reduce the prescription dose coverage to PTV (approximately by 20% with +2 mm translational error and by 10% with +2° rotational error). Although not so great as in PTV, the dose coverage to GTV also has a certain degree of deterioration (approximately by 8% with +2 mm translational error and by 2% with +2° rotational error). Although no statistically significant difference in plan quality or robustness was observed between the coplanar and non‐coplanar plans after applying the same setup error, the probability of incidence of these errors is higher in non‐coplanar plans. Since the non‐coplanar technique also increases treatment times, it is preferable to use the coplanar planning technique if plan quality is comparable between coplanar and non‐coplanar plans.

Xu et al. used the longitudinal grouping strategy to separate the brain lesions into different groups longitudinally for VMAT planning.^19^ The collimator was rotated to 90° to better conform the MLC leaves to the lesion(s) in each group. Their results showed that using this longitudinal grouping strategy had similar dose coverage to targets, but significantly reduced the dose to normal brain tissue, compared to the conventional ungrouping strategy in VMAT planning for multiple brain metastases. Only single‐isocenter coplanar VMAT plans were made, although the authors claimed that this grouping technique could be applied in non‐coplanar plans. In our study, we only applied the conventional ungrouping strategy in planning. Our results have shown a statistically significant difference in the V12 to the whole brain between the original coplanar and non‐coplanar plans. Notably, although the V12 in our coplanar plans was higher than in non‐coplanar plans, it was still within the clinically acceptable tolerance, and might not increase the probability of radiation necrosis of brain tissue due to the multiple fractionations in SRT. However, in a single fraction SRS treatment, this increased V12 may be more critical in inducing brain necrosis.

In addition to saving clinical time and reducing potential patient setup errors, there is a broader spectrum in selecting the beam delivery systems to treat patients using coplanar plans. For example, many O‐ring type of linacs such as Halcyon (Varian Medical Systems, Palo Alto, Calfornia, USA) and TomoTherapy can only implement coplanar plans. They have been widely used in the treatment of multiple brain metastatic cancers, indicating that the coplanar method has a broader clinical application from the perspective of treatment modality selection. Nevertheless, it is of importance to continue improving the plan quality of coplanar method such as enhancing the dose gradient index to PTV and reducing unnecessary dose to normal brain.

Finally, another novelty in our study was the 3D gamma analysis for the modified plans with hypothetical setup errors, which was seldom conducted in other studies. The 3D gamma pass rate can be used as an extra metric to evaluate the plan robustness. Our results showed that a large rotational error, such as +2°, could make the non‐coplanar plan fail in the gamma test. However, other plans all passed the gamma test (> 90% pass rate). One possible reason for the failure of no‐coplanar plans with the +2° rotational error could be subject to the much steeper dose gradient index to PTV in the non‐coplanar plans. The 3D gamma results further supported the observation that the robustness of coplanar plans was superior to their non‐coplanar counterparts.

There are some limitations in our current work. First, two different dose prescriptions: 40 Gy in five fractions and 42 Gy in seven fractions were used for the plan quality evaluation after applying setup errors. The inconsistence in dose prescription was due to the difference in patient characteristics such as the primary tumor site, number of brain metastases, patient age, as listed in Table S1 in the Supplementary Materials. A comprehensive analysis for a patient cohort of narrowed characteristics with identical dose prescription will be performed in our future work.

According to the AAPM medical physics practice guideline 9.a. for SRS‐SBRT (stereotactic body radiation therapy),^20^ tomographic slice thickness of 1−3 mm is recommended for SBRT applications, and the slice thickness should not exceed 1.25 mm for SRS applications. For dose calculation in treatment planning, the use of isotropic calculation grid size of 2 mm or finer is recommended, and for very small targets, a 1 mm grid may be necessary. The second limitation of our current work is that 2 mm was used in both patient CT simulation as the slice thickness and treatment planning as the dose calculation grid size. Although using a coarser resolution could reduce the accuracy of the plan dose calculation and dose indices for plan evaluation, our choice of 2 mm was due to many practical considerations, such as time, labor, patient expense, and insurance reimbursement. Hopefully, with the improvement of work efficiency and optimization of our clinical workflow, a finer CT slice thickness and a finer dose calculation grid, i.e., 1 mm, could be adopted for SRS applications in our clinic in the future. Another benefit of using the 1 mm dose grid size is the possibility of implementing a 3D gamma analysis with a tighter distance‐to‐agreement value, i.e., 1 mm.

## 5 CONCLUSION

In the original single‐isocenter VMAT plans, the non‐coplanar method exhibited lower (steeper) values of GI to PTV and lower V12 to the whole brain, but the coplanar method yielded a lower *D*_max_ to the optic chiasma. Other dose‐volume plan evaluation metrics were similar for both planning techniques. Translational errors exhibited a greater deterioration on PTV and GTV dose coverage than rotational errors for both planning techniques, but rotational errors showed stronger deteriorations of the gamma pass rate of non‐coplanar plans. Considering dose‐volume plan evaluation metrics, we concluded that both coplanar and non‐coplanar plans were satisfactorily robust and clinically acceptable. By considering the setup uncertainty sources, availability of treatment modalities, and plan quality evaluation results, the single‐isocenter coplanar VMAT planning technique might have a greater potential and a broader clinical application for treating MBM.

## AUTHOR CONTRIBUTIONS

**Xiaohuan Sun**: Methodology; software; data analysis; writing—original draft preparation. **Fada Guan**: Methodology; software; data analysis; resources; writing—original draft preparation. **Qinghui Yun**: Data analysis. **Matthew Jennings**: Software; writing—review and editing. **Simon Biggs**: Software; resources; writing—review and editing. **Zhongfei Wang**: Data analysis. **Wei Wang**: Data analysis. **Te Zhang**: Data analysis. **Mei Shi**: Conceptualization; writing—review and editing; project administration. **Lina Zhao**: Conceptualization; methodology; resources; writing—review and editing; project administration; funding acquisition.

## CONFLICT OF INTEREST STATEMENT

Simon Biggs is the founder of Radiotherapy AI Pty Ltd. Other authors report there are no conflict of interest to declare.

## ETHICS STATEMENT

The study was conducted in accordance with the Declaration of Helsinki and approved by the Ethics Committee of Xijing Hospital of Air Force Medical University (protocol code KY20212133‐F‐1, and the date of approval was November 10, 2021).

## Supporting information



Supporting Information

## Data Availability

The authors confirm that the data supporting the findings of this study are available within the article and its supplementary materials.
