# Supplementary material for: Impact of setup errors on the robustness of linac‐based single‐isocenter coplanar and non‐coplanar VMAT plans for multiple brain metastases
Source: J Appl Clin Med Phys. 2024 Mar 4;25(7):e14317. doi: 10.1002/acm2.14317 (PMC11244668; doi:10.1002/acm2.14317)
Supplement: Supplementary file 1 — Supporting Information [file ACM2-25-e14317-s001.pdf]

Supplementary Materials

Table S1. Summary of patient characteristics

Patient characteristics	Number or value
**Age (median and range), years**	43 (31-63)
**Gender**	
Female	7
Male	8
**Primary tumor**	
Lung	10
Breast	2
Gastric carcinoma	1
Liver cancer	1
Cervical carcinoma	1
**Number of lesions per patient**	
Two sites	2
Three sites	9
Four sites	2
Five sites	2
**Target volume**	
GTV (per lesion) (cm^3), n = 49	4.2 (0.9-22.5)
PTV (per patient) (cm^3), n = 15	23.24 (5.86-55.51)

Table S2. Summary of all hypothetical patient positional errors

Plan type	Translational error (mm)			Rotational error (º)		
	X	Y	Z	yaw	roll	pitch
1	1	1	1			
2	2	2	2			
3				1	1	1
4				2	2	2

Table S3. Comparison of plan quality evaluation metrics between the original coplanar and non-coplanar plans

Structure	Dose-volume parameter	Coplanar plan	Non-coplanar plan	*p* value
PTV	CI	0.88±0.03	0.86±0.01	0.002
PTV	GI	4.89±0.45	4.43±0.49	0.000
GTV	V100%(%)	99.96±0.15	99.78±0.53	0.019
Brain	Mean(Gy)	7.45±2.33	7.43±2.32	0.817
Brain	V4(%)	60.53±18.67	62.72±18.08	0.073
Brain	V12(%)	17.10±8.38	14.79±8.93	0.002
Brain stem	D_{max}(Gy)	13.05±7.43	13.87±6.30	0.177
Eye-L	D_{max}(Gy)	4.18±3.19	4.11±2.55	0.872
Eye-R	D_{max}(Gy)	3.80±2.53	3.38±1.81	0.212
Lens-L	D_{max}(Gy)	2.04±1.58	1.20±0.99	0.822
Lens-R	D_{max}(Gy)	1.81±1.28	1.93±1.05	0.319
Optic nerve-L	D_{max}(Gy)	3.49±2.81	4.13±2.55	0.189
Optic nerve-R	D_{max}(Gy)	3.57±2.59	4.03±1.88	0.210
Optic chiasma	D_{max}(Gy)	4.90±3.44	5.95±3.36	0.041

Notes: PTV (planning target volume), GTV (gross tumor volume), CI (Paddick conformal index), GI (Paddick gradient index), mean(Gy) refers to the mean dose in the specified structure, V100%(%) refers to the volume (%) receiving 100% of the prescription dose, V4(%) refers to the volume (%) receiving dose >= 4Gy, V12(%) refers to the volume (%) receiving dose >=12 Gy, D_{max}(Gy) refers to the maximum dose in the specified structure. All results are expressed in mean±SD. SD stands for standard deviation. P value was from paired t-test. $p<0.05$ indicates there was a significant difference, and colored in red.

Table S4. Comparison of the impacts of translational errors on coplanar and non-coplanar plans

Structure	Dose-volume parameter	+1 mm			+2 mm		
		Variation of coplanar	Variation of non-coplanar	*p* value	Variation of coplanar	Variation of non-coplanar	*p* value
PTV	V100%(%)	-9.42±1.88	-9.00±1.88	0.091	-20.20±4.91	-20.22±3.85	0.970
GTV	V100%(%)	-0.16±0.29	-0.21±0.43	0.403	-7.82±5.20	-7.72±6.19	0.830
Brain	Mean(Gy)	-0.04±0.07	-0.03±0.06	0.075	-0.11±0.13	-0.09±0.12	0.081
Brain	V4(%)	-0.27±0.68	-0.24±0.63	0.508	-0.79±1.31	-0.73±1.26	0.742
Brain	V12(%)	-0.13±0.21	-0.11±0.19	0.151	-0.39±0.44	-0.29±0.41	0.112
Brain stem	D_{max}(Gy)	-0.55±0.79	-0.39±0.72	0.544	-1.08±1.33	-0.66±0.97	0.260
Eye-L	D_{max}(Gy)	-0.18±0.35	-0.07±0.24	0.124	-0.18±0.35	-0.07±0.24	0.124
Eye-R	D_{max}(Gy)	-0.09±0.29	-0.13±0.23	0.475	-0.15±0.52	-0.22±0.37	0.443
Lens-L	D_{max}(Gy)	-0.06±0.25	0.04±0.15	0.148	1.20±1.78	1.54±1.85	0.301
Lens-R	D_{max}(Gy)	0.13±0.35	0.04±0.32	0.278	0.11±0.52	0.03±0.52	0.462
Optic nerve-L	D_{max}(Gy)	0.001±0.30	-0.04±0.36	0.700	0.03±0.51	-0.02±0.80	0.795
Optic nerve-R	D_{max}(Gy)	-0.05±0.21	-0.13±0.32	0.401	-0.06±0.25	-0.24±0.47	0.131
Optic chiasma	D_{max}(Gy)	0.05±0.76	0.02±0.80	0.825	0.16±1.20	-0.02±1.18	0.484

Notes: the variation was the difference of the corresponding quantity between the modified plan and the original plan.

Table S5. Comparison of the impacts of rotational errors on coplanar and non-coplanar plans

Structure	Dose-volume parameter	+1°			+2°		
		Variation of coplanar	Variation of non-coplanar	*p* value	Variation of coplanar	Variation of non-coplanar	*p* value
PTV	V100%(%)	-2.71±3.19	-2.26±3.16	0.060	-8.55±4.90	-9.15±4.76	0.602
GTV	V100%(%)	-0.10±0.33	-0.19±0.81	0.448	-1.88±3.42	-2.25±4.94	0.382
Brain	Mean(Gy)	0.01±0.02	0.01±0.01	0.595	0.04±0.05	0.06±0.22	0.645
Brain	V4(%)	0.03±0.13	0.03±0.11	0.870	0.08±0.23	-0.02±0.50	0.462
Brain	V12(%)	0.02±0.07	0.01±0.06	0.588	0.03±0.14	0.02±0.13	0.538
Brain stem	D_{max}(Gy)	0.05±0.63	0.04±0.54	0.900	0.43±0.79	0.09±1.13	0.100
Eye-L	D_{max}(Gy)	0.005±0.42	-0.14±0.39	0.189	0.02±0.85	-0.28±0.64	0.139
Eye-R	D_{max}(Gy)	-0.23±0.38	-0.01±0.44	0.161	-0.23±0.56	0.06±0.81	0.248
Lens-L	D_{max}(Gy)	-0.06±0.49	0.03±0.44	0.460	-0.06±0.13	0.03±0.44	0.460
Lens-R	D_{max}(Gy)	0.16±0.46	0.10±0.25	0.520	0.46±0.81	0.15±0.30	0.146
Optic nerve-L	D_{max}(Gy)	-0.09±0.21	-0.24±0.40	0.109	-0.06±0.36	-0.38±0.56	0.059
Optic nerve-R	D_{max}(Gy)	0.06±0.40	0.06±0.31	0.987	0.42±0.88	0.29±0.70	0.551
Optic chiasma	D_{max}(Gy)	-0.02±0.73	0.05±0.78	0.401	0.10±1.13	0.15±1.25	0.592

Table S6. Comparison of 3D gamma pass rate (3%/2mm) of coplanar and non-coplanar VMAT plans with setup errors

	Coplanar	Non-coplanar
+2 mm translational error	90.35±2.08	90.46±1.76
+2º rotational error	90.14±5.49	87.40±6.89

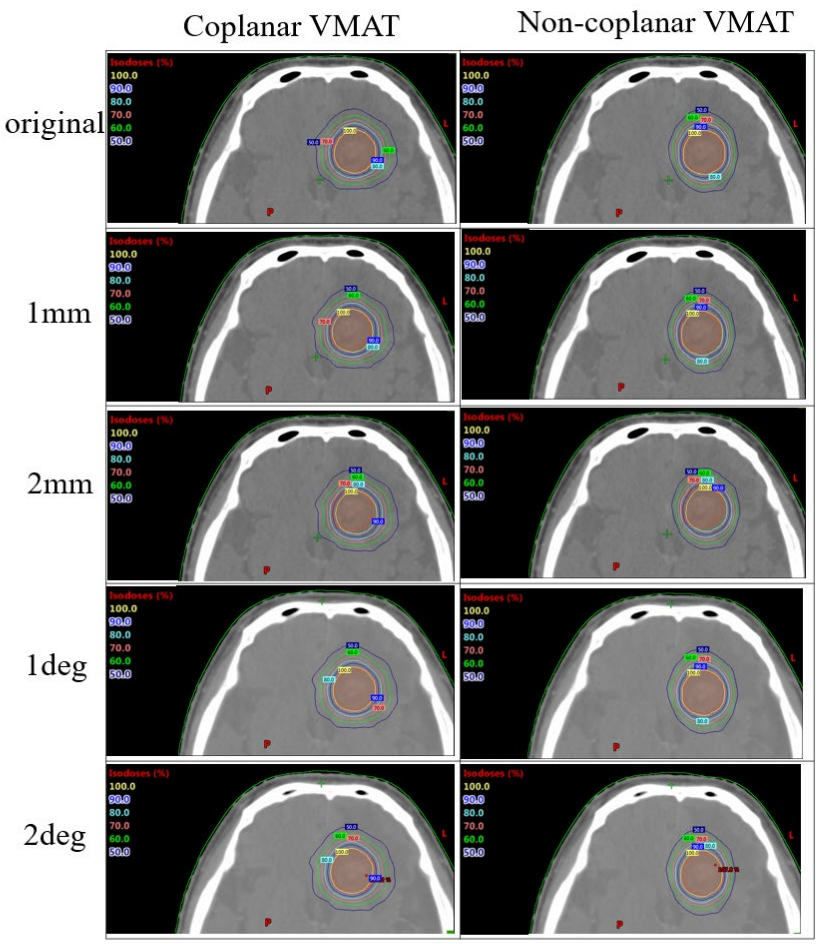

Figure S1. Comparison of single-isocenter VMAT coplanar and non-coplanar plans. Isodose lines from a sample slice of the patient #8’s plans were selected. Original plans, modified plans with +1 mm and +2 mm translational error, and modified plans with +1° and +2° rotational error were depicted.

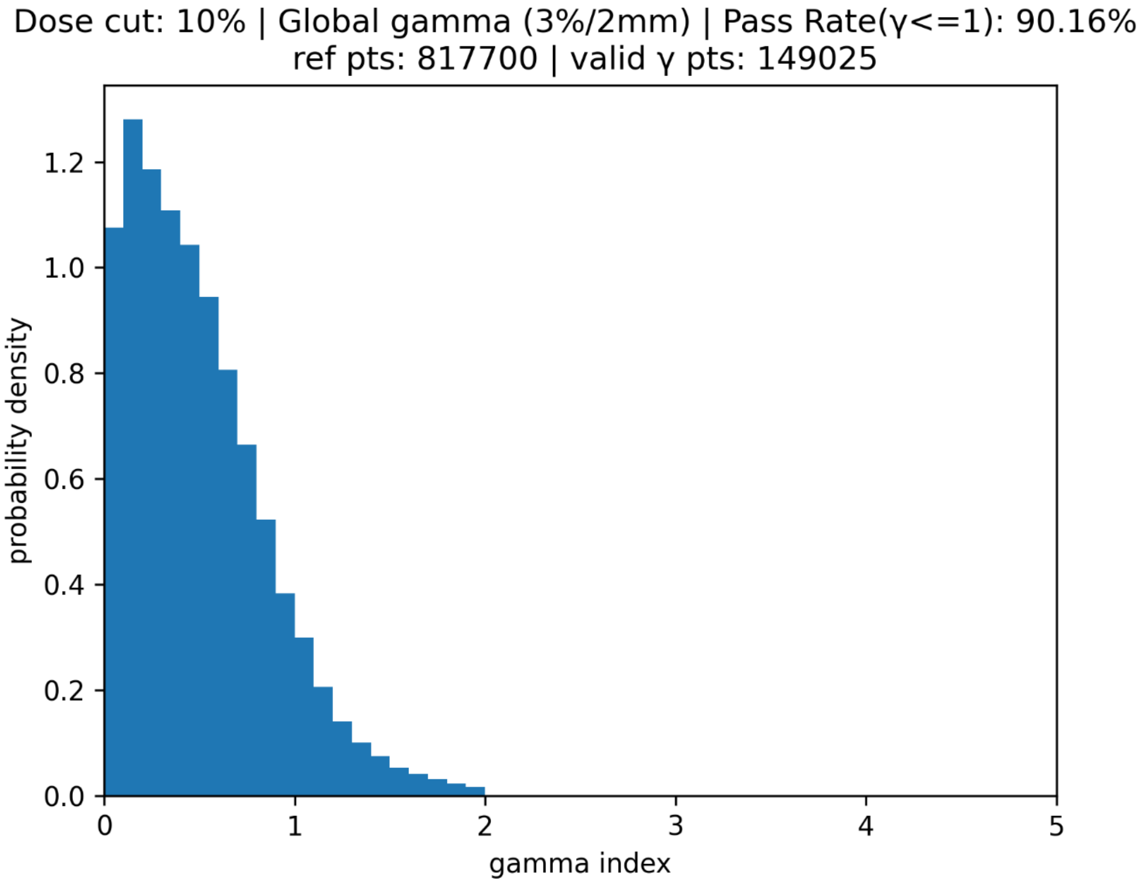

Figure S2. Histogram of gamma index of the patient #8's coplanar plan with +2 mm translational error.

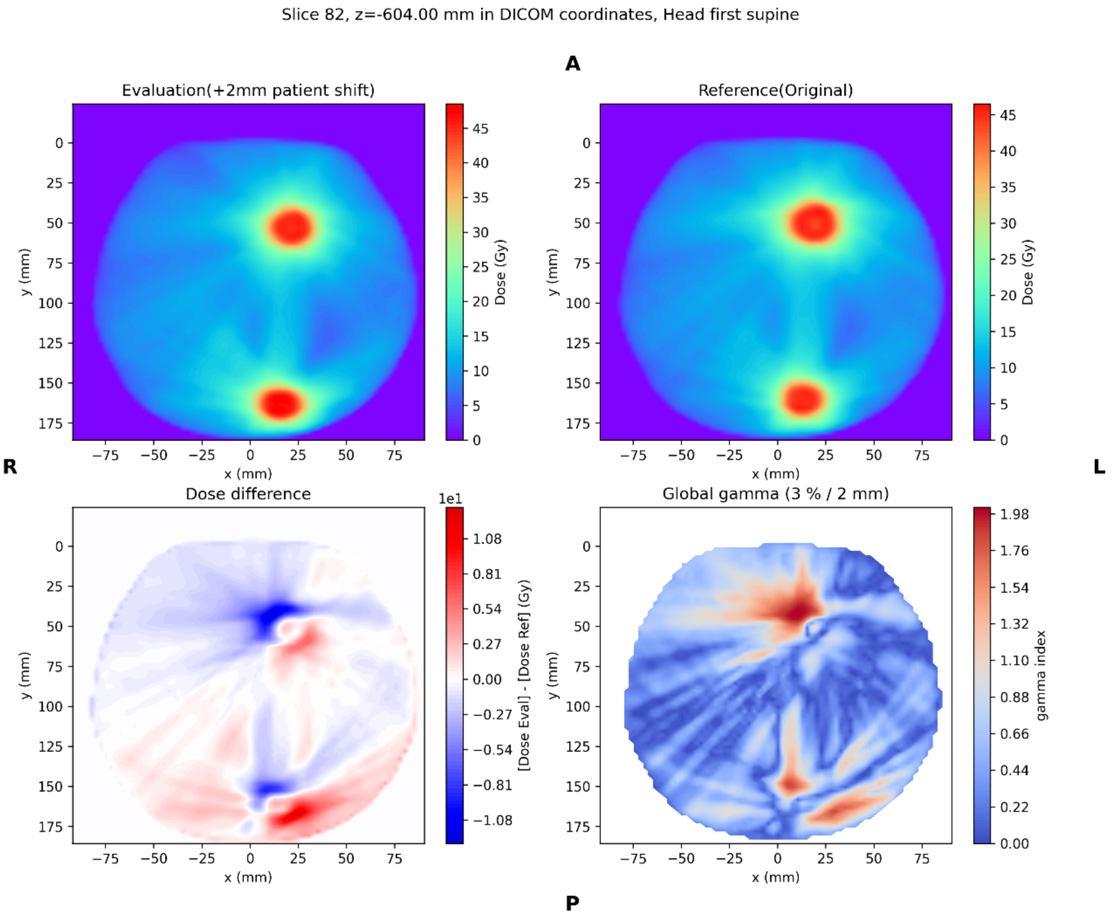

Figure S3. Gamma analysis result for a sample slice of the patient #8's coplanar plan with +2 mm translational error. The dose maps of the original plan and the modified plan as well as the dose difference map were depicted. The global gamma (3%/2mm) map was also depicted.

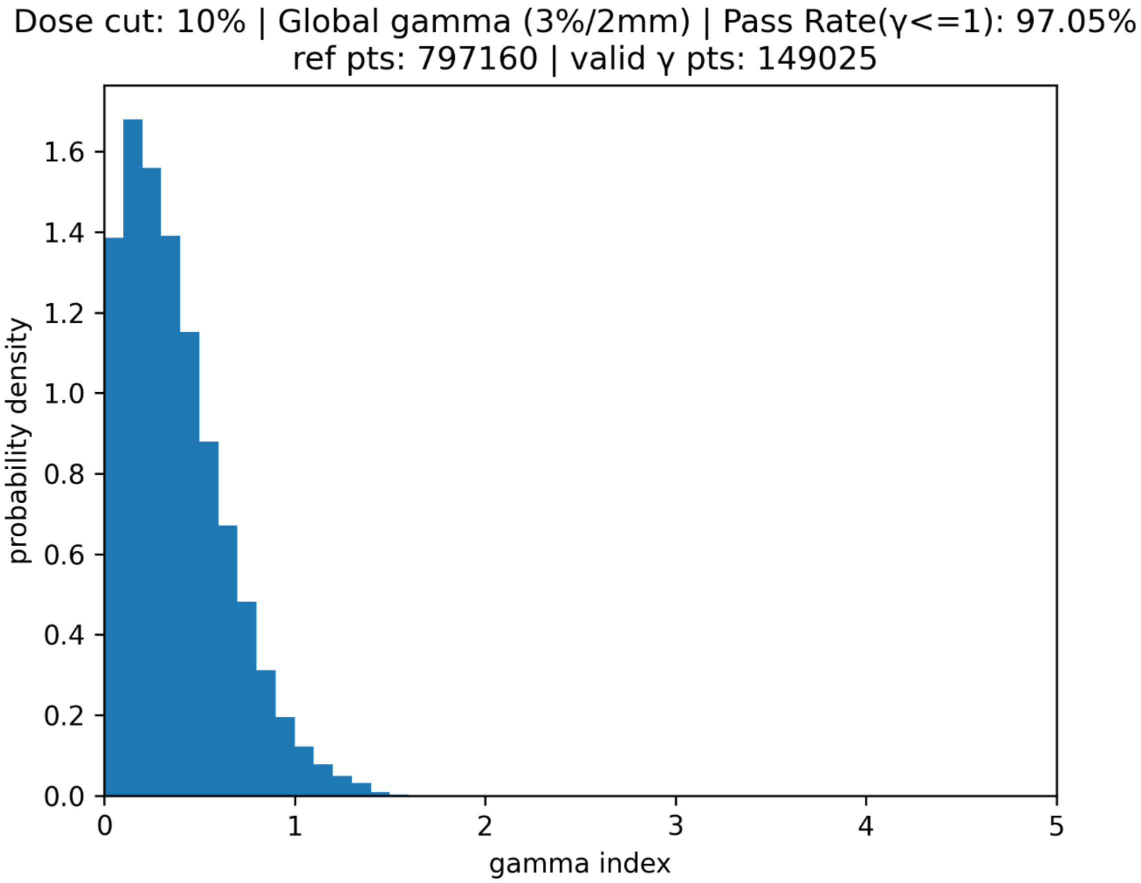

Figure S4. Histogram of gamma index of the patient #8's coplanar plan with +2° rotational error.

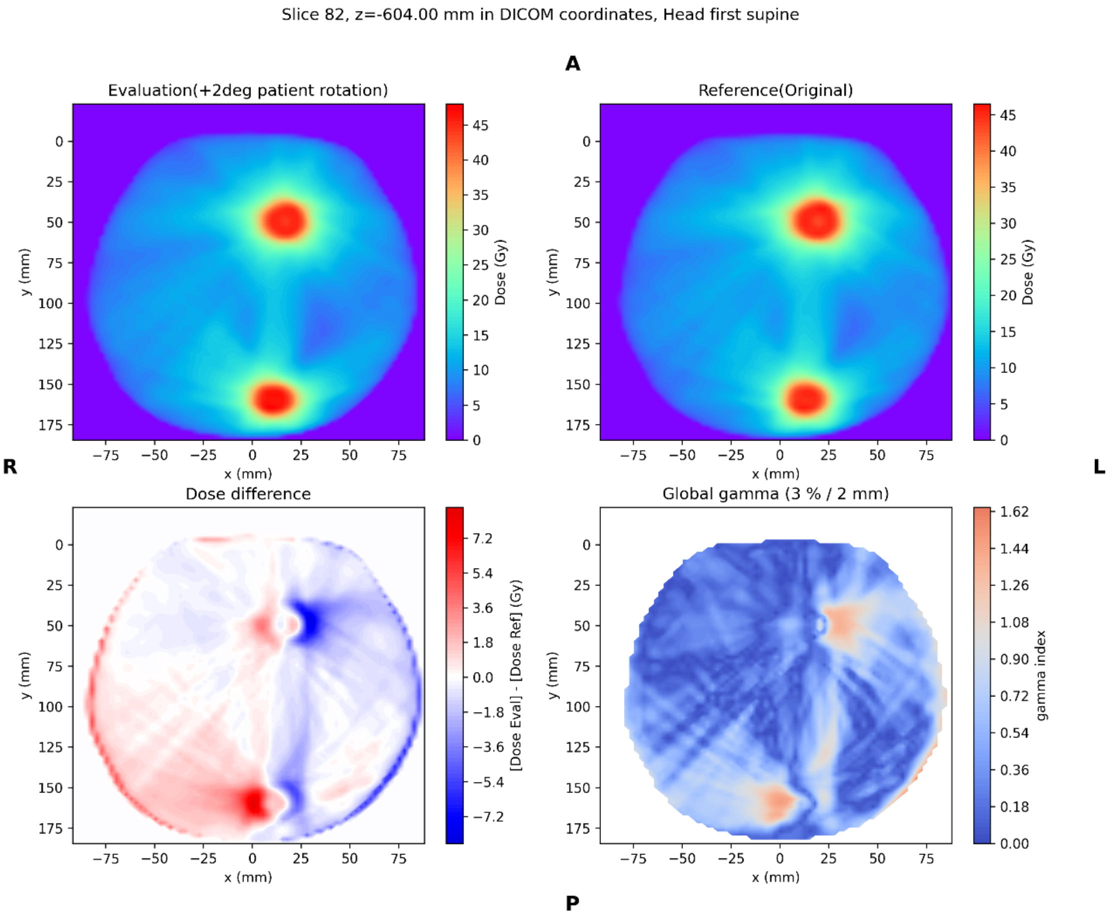

Figure S5. Gamma analysis result for a sample slice of the patient #8's coplanar plan with +2° rotational error. The dose maps of the original plan and the modified plan as well as the dose difference map were depicted. The global gamma (3%/2mm) map was also depicted.

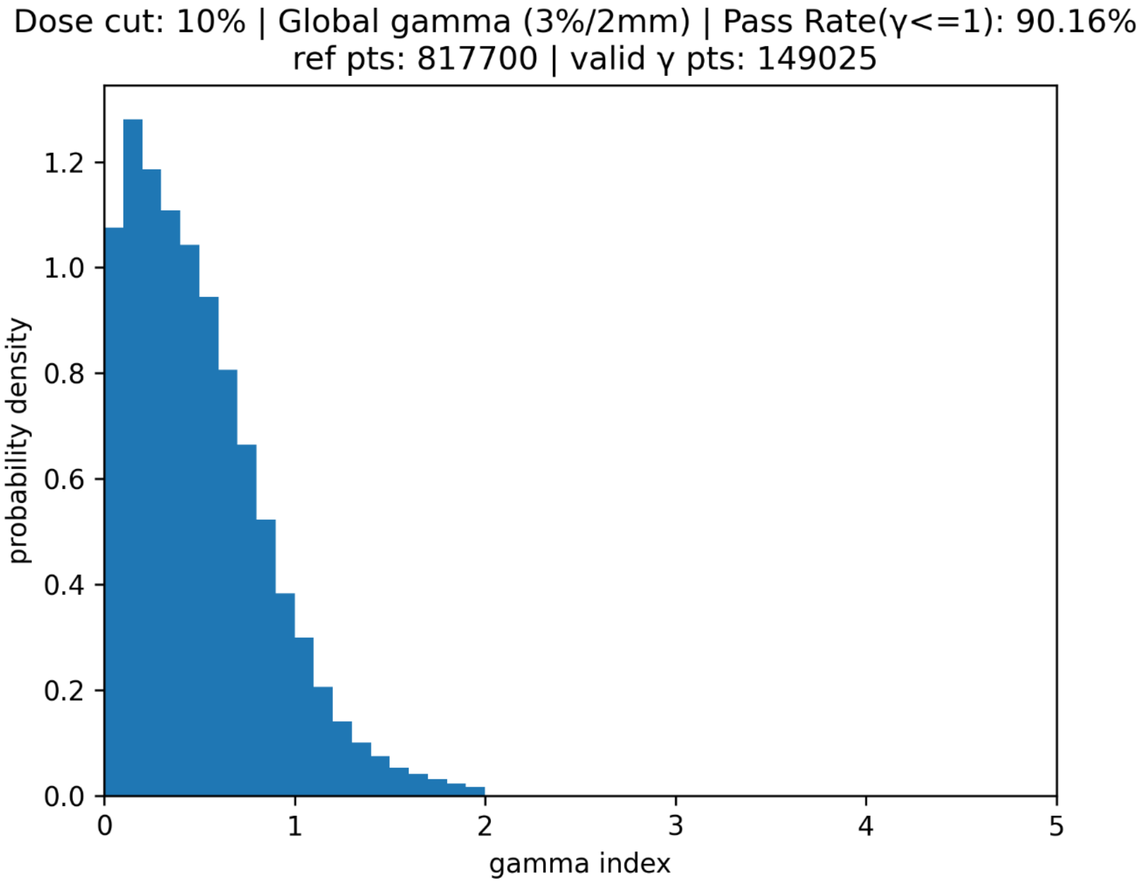

Figure S6. Histogram of gamma index of the patient #8's non-coplanar plan with +2 mm translational error.

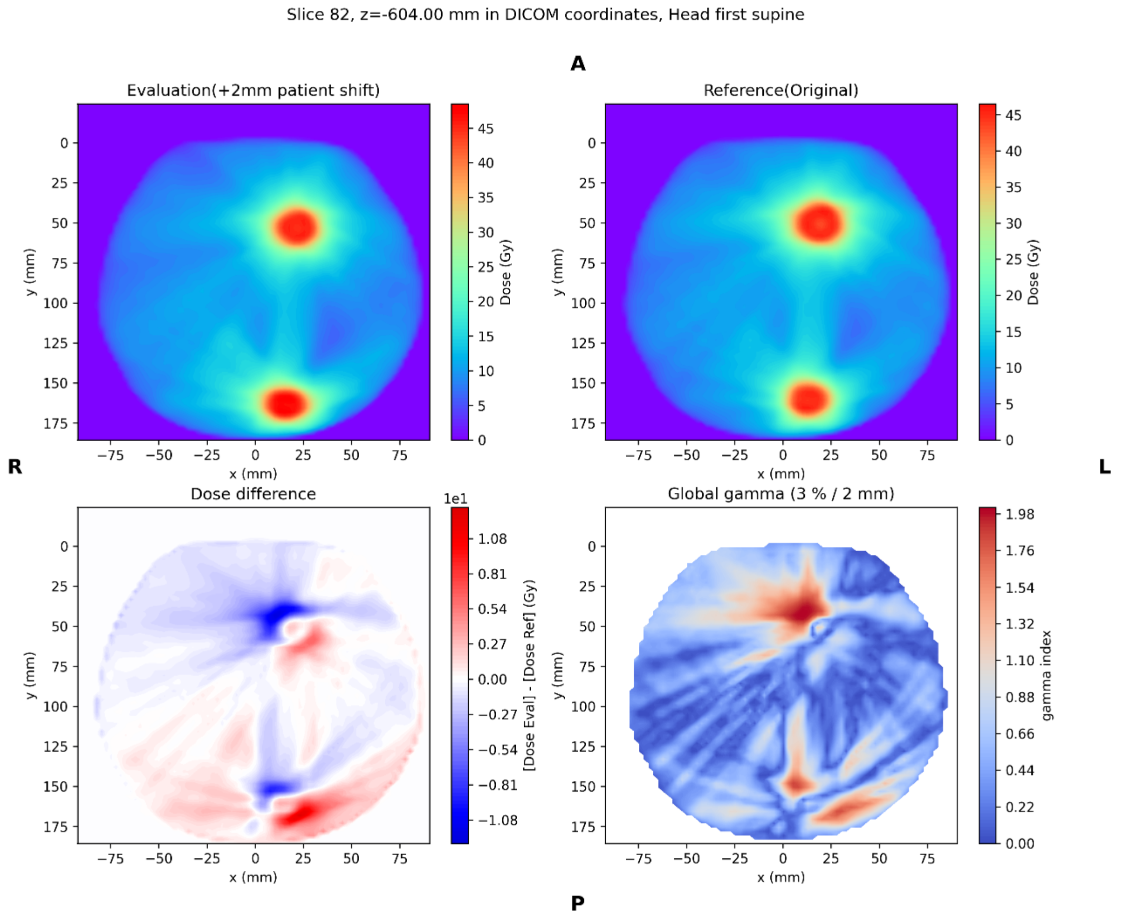

Figure S7. Gamma analysis result for a sample slice of the patient #8's non-coplanar plan with +2 mm translational error. The dose maps of the original plan and the modified plan as well as the dose difference map were depicted. The global gamma (3%/2mm) map was also depicted.

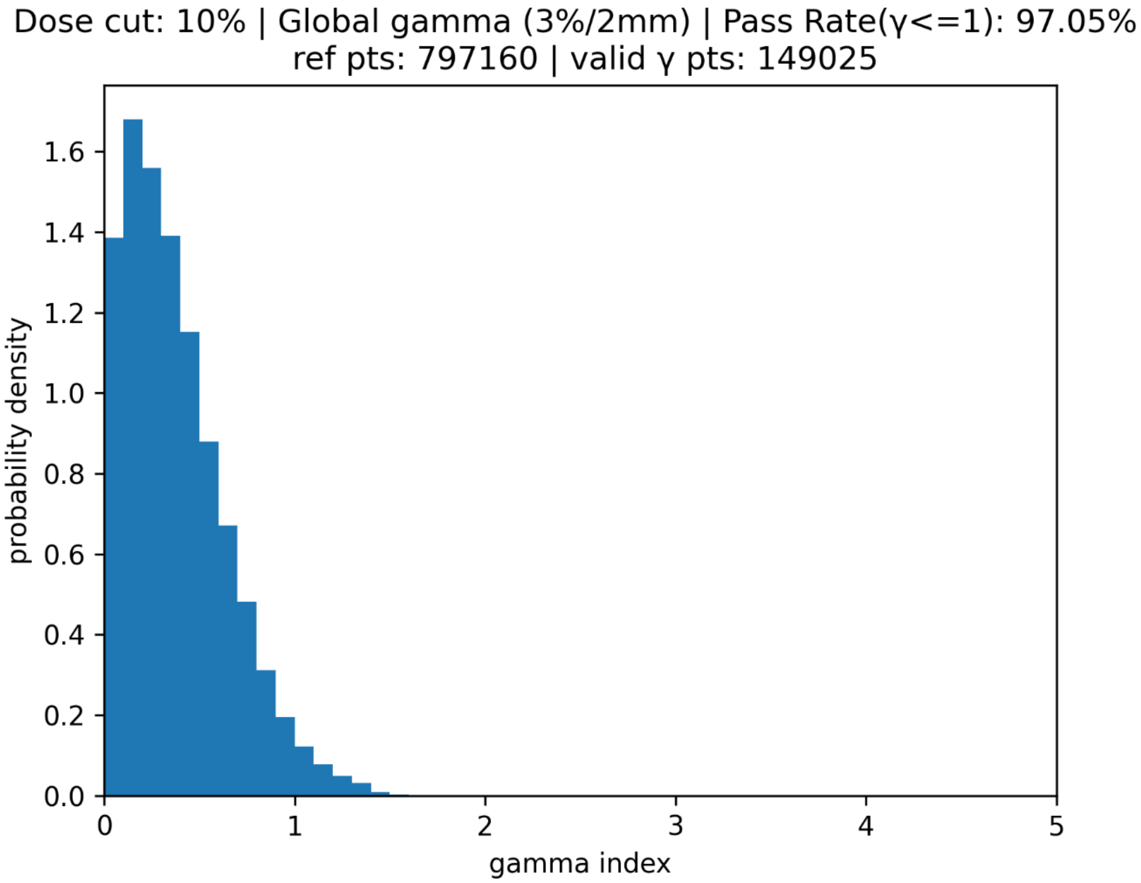

Figure S8. Histogram of gamma index of the patient #8's non-coplanar plan with +2° rotational error.

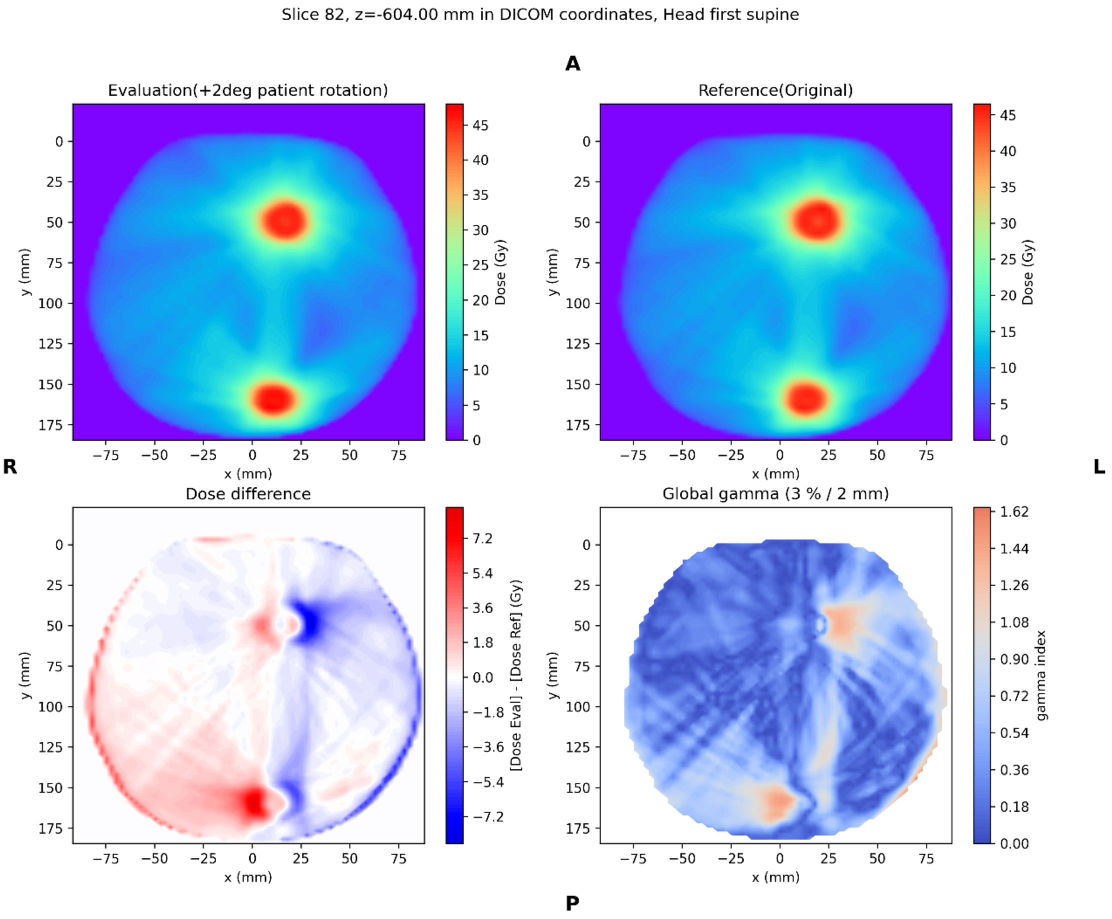

Figure S9. Gamma analysis result for a sample slice of the patient #8's non-coplanar plan with +2° rotational error. The dose maps of the original plan and the modified plan as well as the dose difference map were depicted. The global gamma (3%/2mm) map was also depicted.
